# Osteoporosis, spinal degenerative disorders, and their association with low back pain, activities of daily living, and physical performance in a general population

**DOI:** 10.1038/s41598-024-64706-0

**Published:** 2024-07-09

**Authors:** Shoei Iwata, Hiroshi Hashizume, Noriko Yoshimura, Hiroyuki Oka, Hiroki Iwahashi, Yuyu Ishimoto, Keiji Nagata, Masatoshi Teraguchi, Ryohei Kagotani, Takahide Sasaki, Sakae Tanaka, Munehito Yoshida, Hiroshi Yamada

**Affiliations:** 1 Department of Orthopaedic Surgery, Wakayama Medical University, Wakayama City, Wakayama Japan; 2 Department of Orthopaedic Surgery, Wakayama Rosai Hospital, Wakayama City, Wakayama Japan; 3 School of Health and Nursing Science, Wakayama Medical University, Wakayama City, Wakayama Japan; 4 Department of Preventive Medicine for Locomotive Organ Disorders, Faculty of Medicine, 22nd Century Medical and Research Center, The University of Tokyo, Bunkyo-Ku, Tokyo, Japan; 5 Division of Musculoskeletal AI System Development, Graduate School of Medicine, The University of Tokyo, Bunkyo-Ku, Tokyo, Japan; 6 Department of Orthopedic Surgery, Faculty of Medicine, The University of Tokyo, Bunkyo-Ku, Tokyo, Japan; 7 Department of Orthopedic Surgery, Sumiya Orthopedic Hospital, Wakayama City, Wakayama Japan

**Keywords:** Vertebral fractures, Osteoporosis, Low back pain, Physical performance, Aging, Musculoskeletal disorders, Diseases, Health care, Medical research, Risk factors

## Abstract

Osteoporosis, vertebral fractures, and spinal degenerative diseases are common conditions that often coexist in older adults. This study aimed to determine the factors influencing low back pain and its impact on activities of daily living (ADL) and physical performance in older individuals with multiple comorbidities. This cross-sectional study was part of a large-scale population-based cohort study in Japan, involving 1009 participants who underwent spinal magnetic resonance imaging (MRI) to assess cervical cord compression, radiographic lumbar spinal stenosis, and lumbar disc degeneration. Vertebral fractures in the thoracolumbar spine were evaluated using sagittal MRI with a semi-quantitative method. Bone mineral density was measured using dual-energy X-ray absorptiometry. Low back pain, Oswestry Disability Index (ODI), and physical performance tests, such as one-leg standing time, five times chair-stand time, maximum walking speed, and maximum step length, were assessed. Using clinical conditions as objective variables and image evaluation parameters as explanatory variables, multiple regression analysis showed that vertebral fractures were significantly associated with low back pain and ODI. Vertebral fractures and osteoporosis significantly impacted physical performance, whereas osteoporosis alone did not affect low back pain or ODI. Our findings contribute to new insights into low back pain and its impact on ADL and physical performance.

## Introduction

Many countries, especially developed ones, have been facing rapid population aging^1,2^. Declining musculoskeletal health is a significant concern as it can be an initiating factor for other systemic problems. Studies have shown that musculoskeletal disorders not only cause pain and disability but also lead to secondary health issues such as cardiovascular diseases and mental health disorders due to reduced physical activity and chronic pain^3,4^. Additionally, musculoskeletal disorders are among the most prevalent health conditions worldwide, affecting millions of people and placing a substantial burden on healthcare systems^3,4^. Furthermore, older individuals generally have multiple coexisting conditions. For example, limited to spinal disease, include osteoarthritis, cervical and lumbar spinal stenosis, and osteoporotic vertebral fracture often coexist in the elderly^5^. These coexisting conditions can complicate treatment and management, making it even more critical to address musculoskeletal health proactively^6^.

Therefore, establishing a strategy for the early treatment and prevention of musculoskeletal diseases to improve the healthy life expectancy of people is crucial. In addition, understanding and addressing musculoskeletal decline in the geriatric population are essential for promoting healthy aging, improving the quality of life (QOL) for older individuals, and managing the associated healthcare challenges^4,7,8^. In musculoskeletal disorders, osteoporosis and spinal degenerative disorders are important clinical conditions affecting older individuals and are the focus of our current study. There have been reports indicating that adult sagittal malalignment influences the stresses and loads on lumbar radiographic degenerative changes, which may interact with low back pain^9^. Additionally, studies have shown that low back pain and decreased walking ability are independently associated with severe vertebral fractures, and decreased walking ability is associated with multiple vertebral fractures in women^10^. Therefore, it is expected that the presence of osteoporosis itself is associated with low back pain, as osteoporosis increases the risk of vertebral compression fractures. On the other hand, osteoporotic vertebral fractures result in low back pain and affect individuals’ activities of daily living (ADL) and QOL^11–14^. Older individuals generally have multiple coexisting conditions. Although the prevalence of these conditions and their association with clinical symptoms have been previously reported^15–19^, the influence of comorbid conditions on clinical symptoms remains unknown. Furthermore, no large cohort studies have examined which of these spinal degenerative disorders has the greatest impact.

In 2008, we initiated “the Wakayama Spine Study,” a population-based cohort study aimed at, determining the prevalence of low back pain, osteoporosis, and spinal degenerative diseases, and assessing their impact on ADL/QOL in the general population. The Wakayama Spine Study facilitated the diagnosis of osteoporosis, vertebral fractures, and cervical and lumbar spinal stenosis, thereby allowing us to address the aforementioned objectives. This study aimed to evaluate the coexistence of osteoporosis and spinal degenerative disorders in the general population, and to determine the actual factors influencing low back pain and its impact on ADL and physical performance in older individuals with multiple comorbidities. It is important to note that addressing osteoporosis and spinal degeneration often requires a multidisciplinary approach, which may include lifestyle modification, physical therapy, pharmacotherapy, and surgical intervention. We hope that the results of our study will provide valuable insights to benefit patients in need.

## Results

Table 1 shows the characteristics of the 1011 participants, including their ages and anthropometric measurement data obtained in this study. The mean age and prevalence of low back pain did not differ significantly between men and women; however, the body mass index (BMI) was significantly lower in women than in men. Among the image evaluation parameters, cervical cord compression, vertebral fractures, and osteoporosis were significantly different between the sexes (all *P* < 0.05), whereas radiographic lumbar spinal stenosis.

**Table 1:** Characteristics of participants included in this study stratified by sex.

	Total	Men	Women
	(*N* = 1009)	(n = 335)	(n = 674)
Age (yr)	66.3 ± 13.6	67.3 ± 13.8	65.9 ± 13.4
BMI (kg/m^2^)	23.3 ± 3.6	23.7 ± 3.5*	23.1 ± 3.6
Image evaluation parameters			
Cervical cord compression (%)	23.6	29.3*	21.9
rLSS (%)	28.7	30.3	29.7
LDD	18.0 ± 2.5	17.9 ± 2.4	18.1 ± 2.6
Vertebral fractures	2.1 ± 1.7	2.3 ± 1.5*	2.0 ± 1.8
Osteoporosis (%)	24.9	14.3*	30.3
Clinical conditions			
Low back pain (%)	40.1	36.6	41.8
ODI (%)	12.6 ± 14.4	36.6	13.2 ± 14.9
Physical performance tests			
OLS (s)	35.7 ± 23.9	35.7 ± 24.6	35.7 ± 23.8
CST (s)	8.87 ± 4.0	8.78 ± 3.4	8.91 ± 4.2
Maximum walking speed (s)	3.90 ± 1.4	3.64 ± 1.06*	4.02 ± 1.6
Maximum step length (cm)	64.4 ± 11.1	70.7 ± 10.8*	61.2 ± 11.3

Values are presented as the mean ± SD.

BMI, body mass index; CST, five times chair-stand time; LDD, lumbar disc degeneration; ODI, Oswestry Disability Index; OLS, one-leg standing time; rLSS, radiographic lumbar spinal stenosis.

*P < 0.05 versus women, Student’s *t*-test.

Multiple regression analysis or multiple logistic regression analysis was performed with low back pain, Oswestry Disability Index (ODI), and physical performance tests as objective variables and image evaluation parameters (all predictors in the left column in Tables 2, 3 and 4) as explanatory variables. After adjusting for sex, age, BMI, and other image evaluation predictors (in Model 3), rLSS, LDD, and vertebral fractures were found to be associated with low back pain (*P* = 0.0034, 0.0027, and 0.0321, respectively) but not with cervical cord compression and osteoporosis. Similarly, cervical cord compression and vertebral fractures were associated with the ODI (*P* = 0.0119 and 0.0066, respectively). In addition, the same multiple logistic regression analysis was performed for physical performance tests. No parameter was significantly associated with one-leg standing time (OLS). Five times chair-stand time (CST) was significantly associated with cervical cord compression and vertebral fractures (*P* = 0.0021 and P = 0.0080, respectively). Four of the five image evaluation parameters (cervical cord compression, LDD, vertebral fractures, and osteoporosis) were significantly associated with 6-m walking time at a maximal pace (maximum walking speed) prolongation (*P* = 0.0001, 0.0270, 0.0016, and 0.0144, respectively). Furthermore, cervical cord compression and osteoporosis were associated with a decrease in step length at a maximal pace (maximum step length) (*P* = 0.0004 and 0.0272, respectively).

**Table 2:** Relationship between osteoporosis/spinal degenerative changes and low back pain.

Predictors	Model 1			Model 2			Model 3		
	OR	95% CI	*P*	OR	95% CI	*P*	OR	95% CI	*P*
Cervical cord compression	1.31	0.97–1.76	0.0742	1.27	0.94–1.73	0.1210	1.25	0.91–1.71	0.1743
rLSS	1.74	1.32–2.30	< 0.0001*	1.67	1.25–2.24	0.0006*	1.57	1.16–2.11	0.0034*
LDD	1.12	1.07–1.18	< 0.0001*	1.11	1.05–1.18	0.0005*	1.10	1.03–1.17	0.0027*
Vertebral fractures	1.13	1.05–1.22	0.0013*	1.13	1.04–1.24	0.0043*	1.11	1.01–1.21	0.0321*
Osteoporosis	1.22	0.92–1.63	0.1718	1.32	0.94–1.85	0.1116	1.26	0.88–1.81	0.2085

Multiple regression analysis was performed with low back pain as objective variables and image evaluation parameters (all predictors in the left column) as explanatory variables.

Model 1: Unadjusted.

Model 2: Adjusted for sex, age, and body mass index.

Model 3: Adjusted for the other predictors (cervical cord compression, rLSS, LDD, vertebral fractures, osteoporosis) and sex, age, and body mass index.

CI, confidence interval; LDD, lumbar disc degeneration; rLSS, radiographic lumbar spinal stenosis.

*P < 0.05.

**Table 3:** Relationship between osteoporosis/spinal degenerative changes and ODI.

Predictors	Model 1			Model 2			Model 3		
	Standardized partial regression coefficient	VIF	*P*	Standardized partial regression coefficient	VIF	*P*	Standardized partial regression coefficient	VIF	*P*
Cervical cord compression	0.174	1	< 0.0001*	0.098	1.06	0.0019*	0.081	1.08	0.0119*
rLSS	0.175	1	< 0.0001*	0.059	1.09	0.0651	0.043	1.11	0.1833
LDD	0.208	1	< 0.0001*	0.048	1.89	0.1512	0.023	1.21	0.4945
Vertebral fractures	0.260	1	< 0.0001*	0.097	1.29	0.0053*	0.097	1.34	0.0066*
Osteoporosis	0.122	1	0.0003*	0.013	1.34	0.7210	0.007	1.38	0.8395

Multiple logistic regression analysis was performed with ODI as objective variables and image evaluation parameters (all predictors in the left column) as explanatory variables.

We examined multicollinearity between explanatory variables using VIF (variance inflation factor).

Model 1: Unadjusted.

Model 2: Adjusted for sex, age, and body mass index.

Model 3: Adjusted for the other predictors (cervical cord compression, rLSS, LDD, vertebral fractures, osteoporosis) and sex, age, and body mass index.

CI, confidence interval; LDD, lumbar disc degeneration; ODI, Oswestry Disability Index; rLSS, radiographic lumbar spinal stenosis.

*P < 0.05.

**Table 4:** Relationship between osteoporosis/spinal degenerative changes and physical performance tests.

	Model 1			Model 2			Model 3		
	Standardized partial regression coefficient	VIF	*P*	Standardized partial regression coefficient	VIF	*P*	Standardized partial regression coefficient	VIF	*P*
OLS									
Cervical cord compression	− 0.179	1	< 0.0001*	− 0.021	1.06	0.3549	− 0.015	1.08	0.5146
rLSS	− 0.228	1	< 0.0001*	− 0.021	1.10	0.3685	− 0.016	1.12	0.5126
LDD	− 0.332	1	< 0.0001*	0.000	1.30	0.9856	0.015	1.33	0.5587
Vertebral fractures	− 0.382	1	< 0.0001*	− 0.047	1.33	0.0687	− 0.043	1.40	0.1072
Osteoporosis	− 0.264	1	< 0.0001*	− 0.043	1.36	0.0936	− 0.040	1.40	0.1335
CST									
Cervical cord compression	0.202	1	< 0.0001*	0.096	1.06	0.0008*	0.089	1.08	0.0021*
rLSS	0.171	1	< 0.0001*	0.028	1.09	0.3381	0.015	1.12	0.6080
LDD	0.208	1	< 0.0001*	0.047	1.29	0.3425	− 0.050	1.32	0.1179
Vertebral fractures	0.302	1	< 0.0001*	0.075	1.32	0.0187*	0.087	1.40	0.0080*
Osteoporosis	0.197	1	< 0.0001*	0.015	1.36	0.6425	0.011	1.39	0.7369
Maximum walking speed									
Cervical cord compression	0.203	1	< 0.0001*	0.104	1.06	0.0002*	0.108	1.08	0.0001*
rLSS	0.165	1	< 0.0001*	0.021	1.09	0.4720	0.002	1.11	0.9522
LDD	0.206	1	< 0.0001*	− 0.051	1.29	0.1009	− 0.069	1.32	0.0270*
Vertebral fractures	0.312	1	< 0.0001*	0.010	1.32	0.0025*	0.102	1.39	0.0016*
Osteoporosis	0.250	1	< 0.0001*	0.088	1.35	0.0052*	0.078	1.40	0.0144*
Maximum step length									
Cervical cord compression	− 0.171	1	< 0.0001*	− 0.092	1.06	0.0003*	− 0.091	1.08	0.0004*
rLSS	− 0.168	1	< 0.0001*	− 0.025	1.09	0.3414	− 0.012	1.11	0.6353
LDD	− 0.226	1	< 0.0001*	0.040	1.29	0.1550	0.042	1.32	0.1424
Vertebral fractures	− 0.238	1	< 0.0001*	− 0.025	1.32	0.3807	− 0.027	1.39	0.3490
Osteoporosis	− 0.272	1	< 0.0001*	− 0.069	1.35	0.0158*	− 0.065	1.40	0.0272*

Multiple logistic regression analysis was performed with physical performance tests as objective variables and image evaluation parameters (all predictors in the left column) as explanatory variables.

We examined multicollinearity between explanatory variables using VIF (variance inflation factor).

Model 1: Unadjusted.

Model 2: Adjusted for sex, age, and body mass index.

Model 3: Adjusted for the other predictors (cervical cord compression, rLSS, LDD, vertebral fractures, osteoporosis) and sex, age, and body mass index.

CST, five times chair-stand time; LDD, lumbar disc degeneration; OLS, one-leg standing time; rLSS, radiographic lumbar spinal stenosis.

*P < 0.05.

## Discussion

This study demonstrated that rLSS, LDD, and vertebral fractures were significantly associated with low back pain, whereas cervical cord compression and vertebral fractures were significantly associated with ODI. However, osteoporosis was not significantly associated with low back pain and low back pain-related ADL disorders, as measured using the ODI in the general population. Several previous studies have investigated the osteoporosis and low back pain. Some basic experimental studies have shown a link between osteoporosis and low back pain^20–23^, and certain clinical studies have suggested that osteoporosis can lead to chronic low back pain^24^. However, the true association between osteoporosis and low back pain in a large population-based cohort remains poorly understood. Our finding that osteoporosis was not independently associated with low back pain in our general population cohort study is novel. In contrast, vertebral fractures were identified as independent factors associated with low back pain and low back pain-related ADL disorders. Previous studies have also reported an association between vertebral fractures and low back pain^25–28^, ADL, and health-related quality of life (HRQoL)^11–14^, which is consistent with our findings.

Furthermore, we measured and evaluated several physical performance indicators of locomotive syndrome, including OLS, CST, maximum walking speed, and maximum step length. We analyzed the relationships between these indices and osteoporosis as well as spinal degenerative disorders (cervical cord compression, rLSS, LDD, and vertebral fractures). In the physical performance test, none of the predictors was associated with OLS, but cervical cord compression and vertebral fractures were associated with CST delay. Cervical cord compression, LDD, vertebral fractures, and osteoporosis were associated with maximum walking speed prolongation, and cervical cord compression and osteoporosis with maximum step length. Focusing on vertebral fractures and osteoporosis, the results indicated that vertebral fractures was significantly associated with CST delay and maximum walking speed prolongation, whereas osteoporosis was significantly associated with maximum walking speed prolongation and step length. In other words, both vertebral fractures and osteoporosis affect physical performance. Stanghelle et al.^29^ emphasized the importance of pain management and exercise interventions in older women with osteoporosis and vertebral fractures, as both pain and walking speed were independently associated with HRQoL, which aligns with our study results. Moreover, by comparing the standardized partial regression coefficient, we estimated that physical performance was more strongly associated with vertebral fractures than with osteoporosis.

In summary, our findings suggest that vertebral fractures influence low back pain, low back pain-related ADL, and physical performance. Several studies have shown a relationship between low back pain and radiographic degenerative changes^30–32^, and some have reported that low bone mineral density is associated with kyphotic deformity. For example, The Framingham Study, which involved spinal computed tomography on approximately 2000 adolescents and young adults, revealed a strong association between low bone density and thoracic kyphosis. It suggested that this factor may be predominantly genetic in origin^33^. In addition, Tanishima et al.^34^ reported that decreased bone density was independently associated with kyphosis in older individuals living in a mountain area. Therefore, kyphotic deformity may be observed in older individuals due to subclinical vertebral fractures caused by low bone density. Furthermore, based on our previous studies^35,36^, which revealed that spinal malalignment, particularly kyphotic deformity, was associated with low back pain, we infer that kyphotic deformity resulting from vertebral fractures causes low back pain, low back pain-related ADL disorders, and poor physical performance. The prevalence of vertebral fractures has been reported as a risk factor for new vertebral fractures^37,38^, and vertebral fractures are associated with mortality and life expectancy^39,40^. Together with our results, these findings highlight the importance of preventing vertebral fractures as a critical clinical concern. Although osteoporosis itself was not associated with low back pain or low back pain-related ADL disorders in this study, early intervention for the treatment of osteoporosis, a condition preceding the development of spinal kyphotic deformities, is crucial. According to Sinaki et al.^41^ individuals with osteoporosis-related kyphosis exhibited significantly higher balance abnormalities than those in the control group, and thoracic hyperkyphosis, in combination with reduced muscle strength, plays a central role in increasing body sway, gait unsteadiness, and the risk of falls in osteoporosis patients. Therefore, improving physical performance is an important factor in preventing vertebral fractures.

This study had some limitations. First, although it included over 1000 participants, they may not fully represent the general population, as they were recruited from only two areas in Japan. However, we compared the anthropometric measurements of the study participants with those of the general Japanese population^42^ and found no significant differences in BMI for either men (BMI: 23.71 [3.41] and 23.95 [2.64] kg/m^2^, *P* = 0.33, respectively) or women (BMI: 23.06 [3.42] and 23.50 [3.69] kg/m^2^, *P* = 0.07, respectively). Second, this was a cross-sectional study, which limited our ability to establish causal relationships between osteoporosis and physical performance. The ongoing Wakayama Spine Study, a longitudinal survey, will provide further insight into these causal relationships. Third, we did not examine the psychosocial factors associated with low back pain. Nevertheless, we believe that this study is superior to previous studies, as it evaluated bone mineral density (BMD) using dual-energy X-ray absorptiometry (DXA) and performed spinal magnetic resonance imaging (MRI) assessments in the general population. In addition, we performed a multidimensional evaluation of low back pain, low back pain-related ADLs, and physical performance.

In conclusion, we found that osteoporosis was not significantly associated with low back pain and related ADL disorders (ODI) and that vertebral fractures were related to low back pain, ADL disturbance, and agility decline. These results suggest that the prevention of vertebral fractures is crucial. These findings contribute to multidimensional research on low back pain, low back pain-related ADLs, and physical performance. Needless to say, it is important to note that addressing osteoporosis and spinal degeneration often requires a multidisciplinary approach, which may include lifestyle modification, physical therapy, pharmacotherapy, and surgical intervention. We hope that the results of our study will provide valuable insights to benefit patients in need. Further investigations, along with follow-up surveys, should be conducted to elucidate the causes of low back pain and related disorders.

## Methods

### Ethical statement

All procedures in this study involving human participants were performed in accordance with the ethical standards of the institutional and national research committee and the 1964 Helsinki Declaration and its later amendments or comparable ethical standards. The study was conducted with the approval of the ethical committees of the University of Tokyo (nos. 1264 and 1326) and Tokyo Metropolitan Institute of Gerontology (no. 5). Informed consent was obtained from all the study participants.

### Participants

This population-based study of degenerative spinal diseases, titled the Wakayama Spine Study, was conducted as a sub-cohort of a large-scale Population-based cohort study named the Research on Osteoarthritis/Osteoporosis Against Disability (ROAD). ROAD is a nationwide prospective study of bone and joint diseases, comprising population-based cohorts from various communities in Japan. A detailed profile of the ROAD study has been previously described^43–46^. The baseline database for this study included clinical and genetic information of 3040 inhabitants (1061 men and 1979 women; mean age: 70.6 years; age range: 23–95 years). Participants were recruited from resident registration listings in three communities with different characteristics: an urban region in Itabashi, Tokyo; a mountainous region in Hidakagawa, Wakayama; and a coastal region in Taiji, Wakayama. The participants completed an interviewer-administered questionnaire consisting of 400 items, including lifestyle information such as occupational career, smoking habits, alcohol consumption, family history, medical history, physical activity, reproductive variables, and health-related QOL. However, not all surveys were utilized in the present study; instead, only essential sections were analyzed.

**Figure 1:**
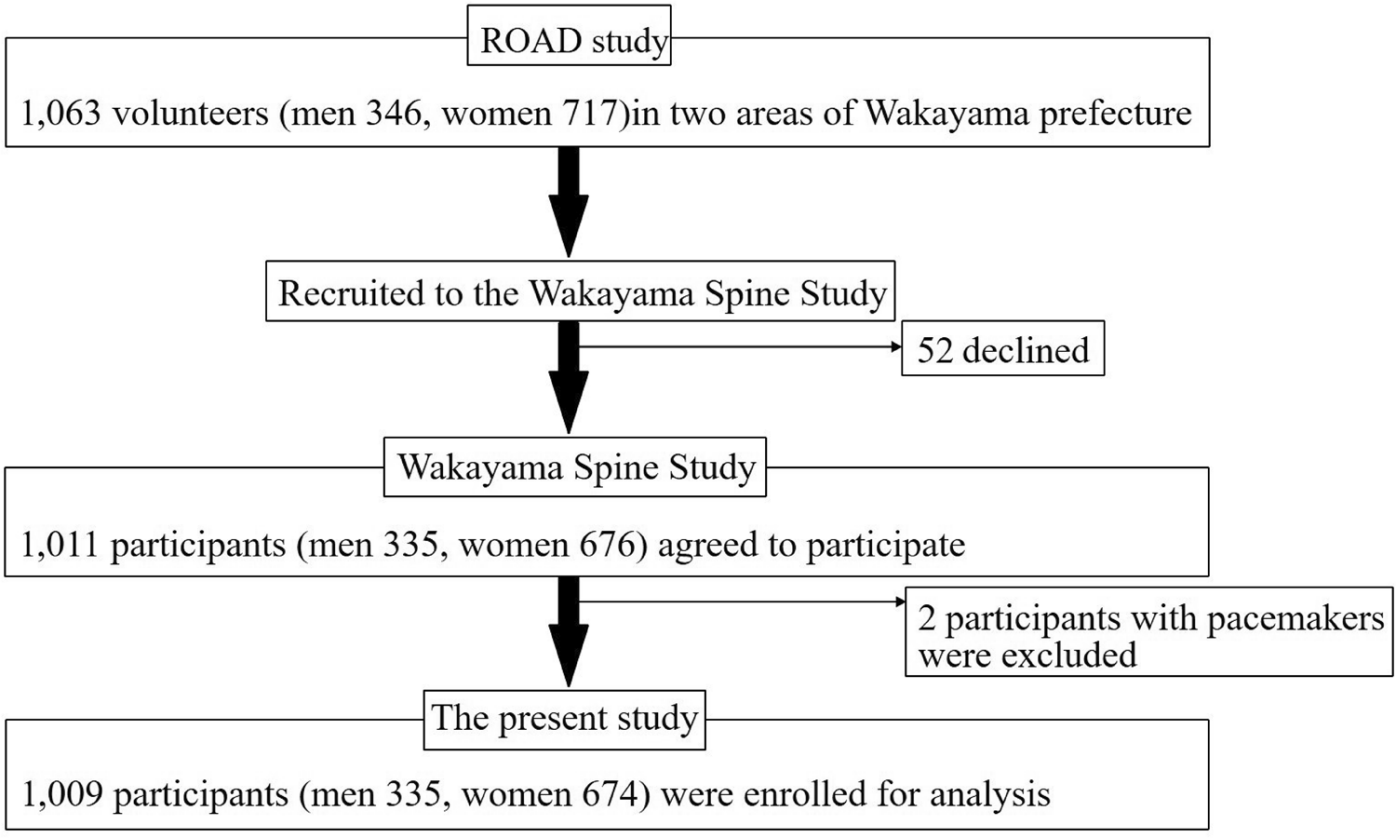
Flow diagram of participant recruitment for the Wakayama Spine Study from the Research on Osteoarthritis/Osteoporosis Against Disability (ROAD) study.

Here, we briefly summarize the profile of the present study. The second visit of the ROAD study began in 2008 and was completed in 2010. All participants in the baseline study were invited to participate in the second visit. In addition to the former participants, inhabitants aged 60 years and older in the urban area and those aged 40 years and younger in the mountainous and coastal areas who were willing to participate in the ROAD survey were also included in the second visit (both the mountainous and coastal areas were in Wakayama prefecture). Finally, 2674 individuals (900 men, 1774 women) participated in the second visit of the ROAD study, comprising 1067 individuals (353 men, 714 women) in the urban area, 742 individuals (265 men, 477 women) in the mountainous area, and 865 individuals (282 men, 583 women) in the coastal area. Among these three communities in the ROAD study, the mountainous and coastal areas, from which we invited all 1607 participants (547 men, 1060 women) to the Wakayama Spine Study, are located in Wakayama prefecture. However, 1063 participants responded to the invitation for the Wakayama Spine Study, and 52 participants declined; therefore, a total of 1011 individuals provided written informed consent and attended the Wakayama Spine Study with MRI examinations. A mobile MRI unit (Excelart 1.5 T, Toshiba, Tokyo, Japan) was used, and a total spinal MRI was performed for all participants on the same day as the clinical examination. Two participants for whom MRI was contraindicated due to the presence of a pacemaker were excluded; thus, 1009 individuals (335 men and 674 women; mean age: 66.3 years; age range: 21–97 years) participated in this study (Fig. 1). BMD was measured at the femoral neck using DXA (with Hologic Discovery DXA system; Hologic, Waltham, MA, USA).

### Definition of low back pain

Experienced board-certified orthopedic surgeons asked all the participants the following question regarding low back pain: “Have you experienced low back pain on most days during the past month, in addition to now?” Those who answered “yes” were defined as having low back pain, as previously described^43,47–50^. Low back pain in this study is defined as pain persisting for at least 1 day, located in the posterior aspect of the trunk, between the 12th rib and the lower border of the gluteal sulcus. It may or may not be accompanied by radiating pain in one or both lower limbs^51^.

### ODI

The ODI, derived from the Oswestry Low Back Pain Questionnaire^52–54^, was used by clinicians and researchers to quantify the level of disability due to low back pain. The patient questionnaire included several questions related to pain intensity, ability to walk, sit, stand, self-care, travel, sexual function, lifting, social life, and sleep quality. Participants selected responses that most closely resembled their symptoms. The index scores ranged from 0 to 100, with “0” indicating no disability and “100” indicating the most severe. In this study, we considered ODI as low back pain-related ADL disorders.

### Evaluation of cervical cord compression

**Figure 2:**
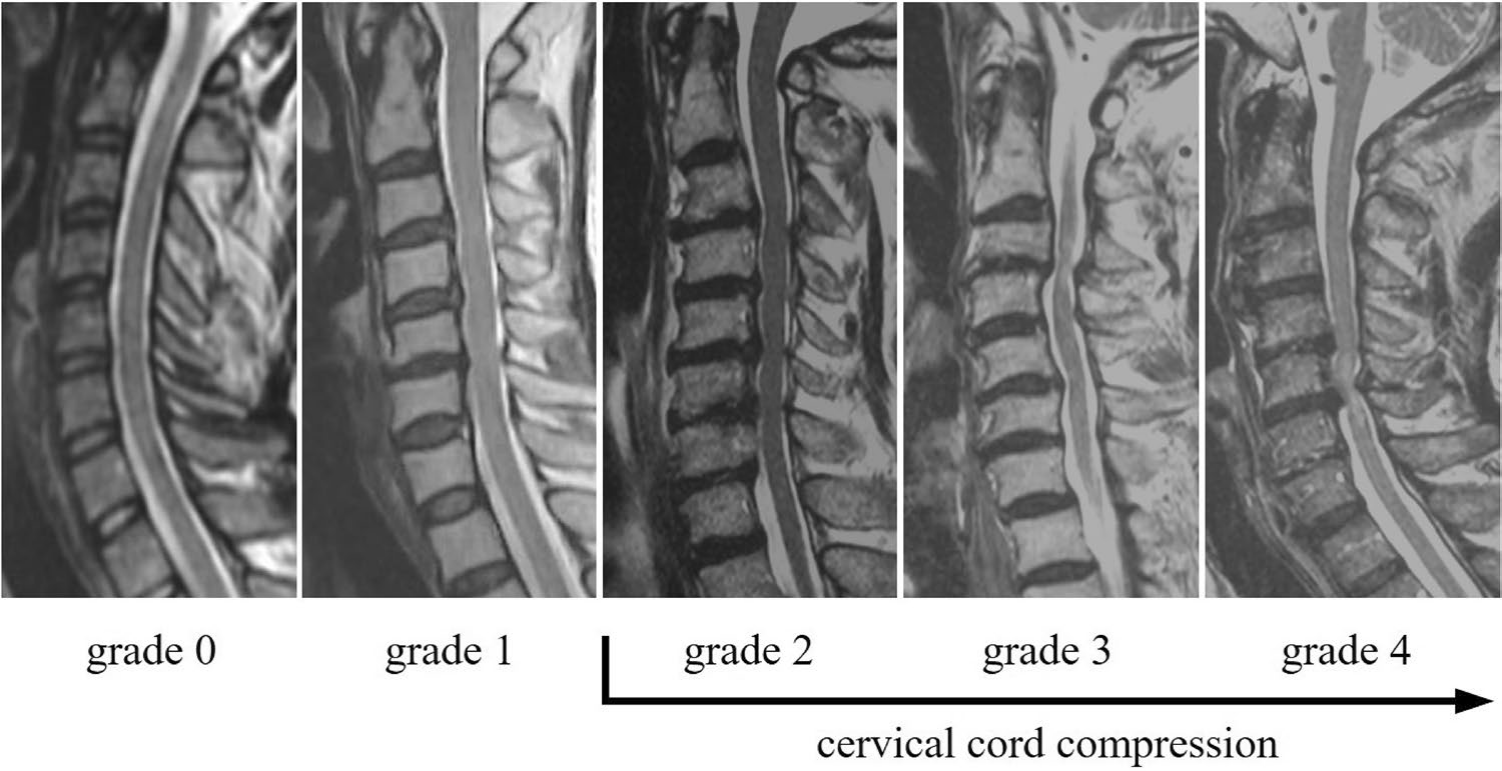
Measurement figures of cervical cord compression. Cervical cord compression was defined as grade 2 or higher at the most severely affected intervertebral disc level.

Cervical cord compression was defined as the compression of the anterior and posterior components of the spinal cord, as previously described^55^. Cervical cord compression was evaluated at each intervertebral level from C2/3 to C7/Th1 and graded as follows: grade 0, no compression of the spinal cord, but the subarachnoid space was present; grade 1, no compression of the spinal cord without the subarachnoid space; grade 2, compression of less than one-third of the spinal cord; grade 3, compression of more than one-third but less than two-thirds of the spinal cord; and grade 4, compression of more than two-thirds of the spinal cord. Cervical cord compression was defined as grade 2 or higher at the most severely affected intervertebral disc level and was analyzed accordingly (Fig. 2). Intra- and inter-observer variabilities for cervical cord compression, evaluated using kappa analysis, were 0.78 and 0.72, respectively, and were deemed sufficient for assessment^55^.

### Evaluation of rLSS

**Figure 3:**
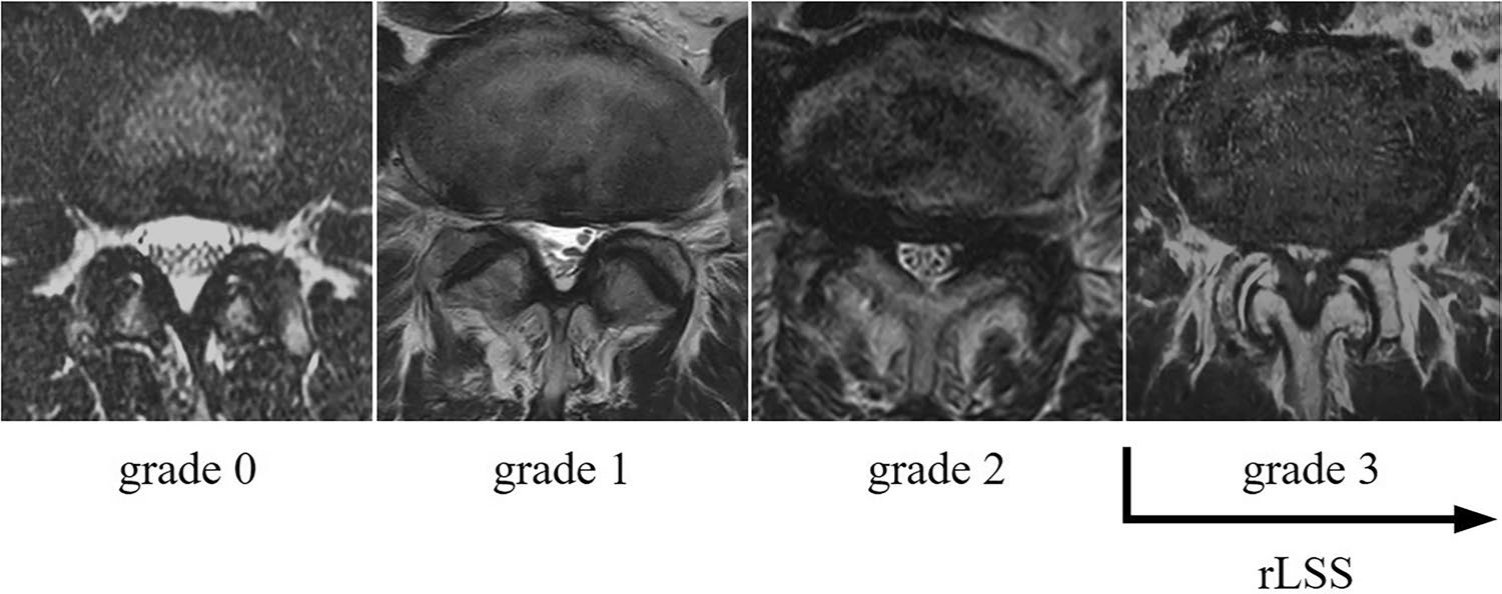
Measurement figures of rLSS (radiographic lumbar spinal stenosis). rLSS was defined as grade 3 at the most severely affected lumbar intervertebral disc level.

The severity of rLSS was assessed using qualitative measurements performed by a well-experienced orthopedic surgeon. Axial images at each lumbar intervertebral level (L1/2 to L5/S1) were obtained from the films, as previously described^56^. The severity of central stenosis was assessed according to general guidelines (Suri classification)^57^. According to the Suri classification, the rLSS was divided into four levels: grade 0, no narrowing; grade 1, mild narrowing of less than one–third of the normal area; grade 2, moderate narrowing of one–third to two–thirds of the normal area; and grade 3, severe narrowing of more than two–thirds of the normal area. rLSS was defined as grade 3 at the most severely affected lumbar intervertebral disc level and was analyzed accordingly (Fig. 3). Intra- and inter-observer variabilities for rLSS, evaluated using kappa analysis, were 0.82 and 0.77, respectively, and were deemed sufficient for assessment^56^.

### Evaluation of LDD

**Figure 4:**
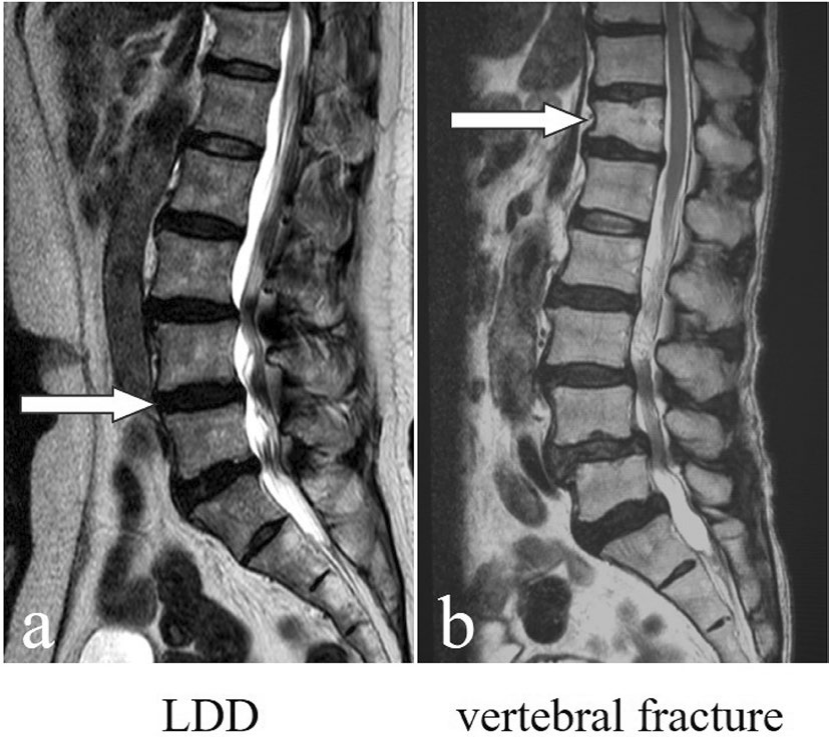
(**a**) Example figure of LDD (lumbar disc degeneration); (**b**) Example figure of vertebral fracture in the thoracolumbar spine.

The degree of LDD on MRI (Fig. 4a) was classified into five grades based on the Pfirrmann classification system^58^, as previously described^59^. The total score (5–25), which was the sum of each intervertebral grade at L1/2–5/S, was used as the index. Intra- and inter-observer variabilities for LDD, evaluated using kappa analysis, were 0.94 and 0.94, respectively, and were deemed sufficient for assessment^59^.

### Evaluation of vertebral fractures in the thoracolumbar spine

Vertebral fractures and their severity were assessed using the Genant semi-quantitative (SQ) method^60^ with sagittal MR images of the Th11-L1 vertebrae (Fig. 4b). The SQ method was graded from 0 to 3, defined as follows: grade 0 = normal; grade 1 = mildly deformed (approximately 20–25% reduction in anterior, middle, and posterior height, and a 10–20% reduction in area); grade 2 = moderately deformed (approximately 25–40% reduction in height and a 20–40% reduction in area); and grade 3 = severely deformed (approximately 40% reduction in height and area). The total score (0–9) was used as an index by summing the respective grades of the Th11-L1 vertebrae. The prevalence of vertebral fractures was reported to be highest in Th11-L1 lesions^10^. To evaluate the intra-observer variability, 100 randomly selected MRIs of the entire spine were rescored by the same observer (MT) more than 1 month after the first reading. Furthermore, to evaluate inter-observer variability, 100 other MRIs were scored by two experienced orthopedic surgeons (MT and RK) using the same SQ method. Intra- and inter-observer variabilities for vertebral fractures, evaluated using kappa analysis, were 0.87 and 0.84, respectively, and were deemed sufficient for assessment.

### Evaluation of osteoporosis

Osteoporosis was defined as BMD < 70% of the peak bone mass, according to the criteria of the Japanese Society for Bone and Mineral Research^61^. BMD at the femoral neck < 0.604 g/cm^2^ in men and < 0.551 g/cm^2^ in women indicated osteoporosis and was analyzed accordingly. In this study, femoral neck osteoporosis was used to assess the incidence of osteoporosis.

### Physical performance tests

Several tests were performed to evaluate physical performance, as previously reported^55,56^. In this study, the following four items were selected: OLS, CST, maximum walking speed, and maximum step length. The OLS for each leg was measured using a stopwatch (upper limit, 60 s), and the mean time for both legs was used for further analysis. OLS appears to be a significant and easy-to-administer predictor of injurious falls reported by a previous study^62^; therefore, the present study adopted it. The time taken for five consecutive chair rises without the use of hands was recorded. CST is a specific test used in assessing functional capacity, particularly focusing on lower limb strength and endurance. This test is commonly employed in populations such as older individuals to evaluate their functional abilities^63,64^. Walking speed was measured by recording the time taken to walk 6 m at the usual pace in a hallway. Similarly, the 6-m walking time at the maximal pace was measured. The participants were provided with a full explanation of each test but were not provided with any training. Walking speed and step length are effective indicators of physical performance, providing valuable information across various contexts. In older individuals, both are associated with fall risk and survival^65,66^. In this current study, we evaluated their maximum values. Finally, these four physical tests were effective for the evaluation of physical performance indicators of the locomotive syndrome, respectively^67^.

### Statistical analyses

Five image evaluation parameters (cervical cord compression, rLSS, LDD, vertebral fractures, and osteoporosis) were analyzed. Six clinical conditions and parameters, namely low back pain, ODI, and four physical performance tests (OLS, CST, maximum walking speed, and maximum step length), were also evaluated. Baseline characteristics between the sexes were compared using a non-paired Student’s *t*-test for numerical variables. Multiple logistic regression analysis was used to estimate the association between clinical conditions (low back pain, ODI, OLS, CST, maximum walking speed, and maximum step length) and image evaluation parameters (cervical cord compression, rLSS, LDD, vertebral fractures, and osteoporosis), after adjusting for age, sex, and BMI (kg/m^2^). Clinical conditions were used as objective variables, whereas image evaluation parameters were used as explanatory variables. All statistical tests were performed at a two-sided significance level of 0.05. Data were analyzed using JMP version 14 (SAS Institute Japan, Tokyo, Japan).

## Data Availability

All data generated or analyzed during this study are available from the corresponding author upon reasonable request.
